# Exogenous Nano-Silicon Treatment Improved the Low-Temperature Tolerance of Rice Seedlings

**DOI:** 10.3390/plants15131983

**Published:** 2026-06-26

**Authors:** Ke Ma, Xin Liu, Zexin Qi, Yuanyuan Zhou, Heping Xu, Yao Ma

**Affiliations:** 1 Agronomy College, Jilin Agricultural Science and Technology College, Jilin 132101, China; 2 Agronomy College, Jilin Agricultural University, Changchun 130118, China

**Keywords:** low temperature stress, rice, nanoparticles, photosynthesis, antioxidant

## Abstract

Silicon plays an important role in enhancing plant tolerance to abiotic stress. However, the differential regulatory effects of ionic silicon (Ion-Si) and silicon nanoparticles (SiNPs) on rice seedlings under low temperature (LT) stress have been less studied. This study aimed to investigate the effects of ionic silicon and silicon nanoparticles on rice growth, photosynthetic performance, carbon metabolism, antioxidant defense, and yield formation under low-temperature stress. The results indicated that low-temperature stress significantly inhibited the growth of rice seedlings. Exogenous application of Ion-Si and SiNPs effectively alleviated LT-induced growth inhibition and promoted the recovery of rice. SiNPs demonstrated a stronger effect than Ion-Si in maintaining seedling growth, particularly in enhancing plant height, root length, leaf area, dry weight, and root activity. Low-temperature stress significantly reduced chlorophyll content and photosynthetic capacity, including net photosynthetic rate, stomatal conductance, transpiration rate, intercellular CO_2_ concentration, and Rubisco activity. However, under LT stress, both Ion-Si and SiNPs increased chlorophyll content, improved photosynthesis, and enhanced Rubisco activity, with SiNPs showing greater improvement in photosynthetic performance compared to Ion-Si. Additionally, silicon application regulated carbohydrate metabolism by increasing soluble sugar content and enhancing the activities of sucrose phosphate synthase and sucrose synthase, thereby promoting osmotic regulation and energy supply. SiNPs had a stronger effect on carbohydrate metabolism and photosynthate transport than Ion-Si. Furthermore, LT stress increased oxidative damage, manifested as elevated levels of H_2_O_2_ and malondialdehyde. Exogenous Ion-Si and SiNPs reduced ROS accumulation and lipid peroxidation by increasing the activities of antioxidant enzymes, including superoxide dismutase, peroxidase, and catalase. Compared with Ion-Si, SiNPs showed a stronger ability to enhance antioxidant defense and alleviate oxidative damage. Application of silicon mitigated yield loss under low temperature stress, and SiNPs was more effective than Ion-Si in maintaining rice yield, mainly by increasing the number of effective panicles, grains per panicle, and seed setting rate. This study revealed the distinct physiological roles of Ion-Si and SiNPs in rice cold tolerance and provided a theoretical foundation for the application of silicon-based fertilizers in rice production under low-temperature conditions.

## 1. Introduction

Rice (*Oryza sativa* L.) is widely cultivated worldwide and provides a major food source for more than half of the global population [1]. As a thermophilic crop originating from tropical and subtropical regions, rice is particularly vulnerable to low-temperature stress [2]. Low-temperature events frequently occur in major rice-growing countries, including the United States, Japan, Korea, and China. With the rapid development of mechanized direct seeding, the likelihood of rice seedlings being exposed to chilling stress has further increased [3]. In the double-season rice production areas of China, early rice is often subjected to “late spring coldness” at the seedling stage, a typical adverse weather event accompanied by low temperature, cloudy conditions, and rainfall. Such stress can severely impair the growth of direct-seeded early rice seedlings, resulting in poor seedling establishment, reduced tillering capacity, delayed reproductive development, and ultimately yield loss [4,5]. Although the sowing date of early rice has tended to advance under global warming, the occurrence of low-temperature events has not decreased. Furthermore, climate change has increased the frequency of extreme and abnormal weather events [6], thereby increasing the risk of “late spring coldness” during the early growth period of rice. Therefore, low-temperature-induced growth suppression and yield reduction in rice remain major concerns in rice production and research [7].

Low-temperature stress is known to promote the excessive accumulation of reactive oxygen species (ROS) in plants, which can damage cell membrane integrity and disrupt physiological and metabolic homeostasis, eventually leading to cell death [8,9]. Antioxidant enzymes, such as superoxide dismutase (SOD), peroxidase (POD), and catalase (CAT), play crucial roles in plant defense responses under stress conditions by efficiently scavenging ROS and mitigating stress-induced injury [8,9]. In addition, low temperature significantly suppresses nitrogen uptake capacity in rice, while nitrogen acquisition and utilization are directly linked to yield performance [10]. With the progression of global warming, rice cultivation in northeast China has expanded substantially, and this region now accounts for over 14% of the national rice output [11]. In addition, approximately 75% of rice production in northeast China is supplied as commercial grain. In recent years, rice-growing areas in China have experienced more frequent extreme temperature events, among which cold damage has become the leading meteorological hazard affecting rice production, particularly in northeast China [12,13]. Low-temperature stress represents a key factor limiting stable rice production in high-latitude regions. Low-temperature stress often results in stunted growth and reduced seedling vigor in rice, thereby enhancing the competitive advantage of weeds. In addition, rice seedlings exposed to low temperatures are more susceptible to herbicide injury during weed control practices [14]. It has been reported that chilling conditions reduce the activity of membrane-bound enzymes, inhibit herbicide metabolism, and ultimately increase herbicide phytotoxicity in rice [15]. Thus, rapid post-stress recovery is critical for rice seedlings to resume normal growth and development, suppress weed interference, and reduce potential yield losses.

Silicon (Si) is mainly taken up by plant roots in the form of monosilicic acid Si(OH)_4_ and is then transported to different tissues through the xylem. During water loss, the concentration of monosilicic acid increases, which promotes its dehydration and polymerization, leading to the formation of hydrated silica deposits SiO_2_·nH_2_O in cell walls, cell lumens, or outer plant structures [16]. Plants have developed an efficient Si transport system mediated by Lsi-type proteins, including *Lsi1*, *Lsi2*, *Lsi3*, and *Lsi6*. The coordinated regulation of these transport proteins at specific subcellular locations, together with their expression patterns, contributes to improved Si uptake and accumulation in plants [17].

Rice is one of the most typical Si-accumulating crops, and the identification of Si influx and efflux transporters, such as *Lsi1* and *Lsi2*, has provided an important physiological basis for understanding Si uptake and transport in rice. These findings indicate that the response of rice to exogenous Si depends not only on Si supply but also on Si absorption, transport, and deposition in plant tissues [18,19,20]. Therefore, studies on different Si sources, including conventional ionic Si and nano-Si materials, are important for improving Si-based stress management in rice.

Nanotechnology offers a sustainable and broadly applicable strategy for controlling viral diseases in plants through multiple mechanisms, including direct antiviral activity, gene silencing, early diagnosis, and enhancement of plant defense responses [21]. Nanomaterials are generally described as materials that possess at least one dimension within the nanoscale range of 1–100 nm or are constructed from nanoscale structural units [22]. Owing to their small size, these materials can pass through plant cell walls and membranes. Previous evidence has shown that silicon nanoparticles (SiNPs, approximately 20 nm) exert stronger effects on plants than ionic silicon (Ion-Si) [23]. The higher effectiveness of SiNPs is mainly attributed to their unique nanoscale characteristics, such as a large specific surface area for nutrient adsorption, improved cellular uptake efficiency, and sustained release behavior [24]. Due to their nanoscale dimensions and large specific surface area, SiNPs can efficiently adsorb nutrient ions, improve crop water absorption, promote the activation and release of available nutrients in soil, and increase the activities of root and rhizosphere enzymes. Together, these effects facilitate the uptake and utilization of nutrients and help plants mitigate abiotic stress [25,26,27]. Previous studies have shown that foliar application of SiNPs can reduce copper toxicity by modulating proline metabolism and antioxidant defense systems [28]. In addition, silicified cells function as physical barriers that limit the invasion of bacteria and other pathogens, stabilize polysaccharide matrix structures, and improve the mechanical strength of leaf tissues [29].

In rice, SiNPs have also been reported to alleviate stress-induced injury. For example, SiNPs reduced cadmium toxicity in rice cells by mitigating oxidative damage and regulating cellular defense responses [30,31]. These results suggest that SiNPs may enhance stress tolerance in rice through the regulation of ROS homeostasis, antioxidant defense, and cellular protection. However, most previous studies have focused on heavy metal toxicity, salt stress, or other abiotic stresses, whereas information on the role of SiNPs in rice under low-temperature stress remains limited.

Current studies indicate that ionic silicon (Ion-Si) can alleviate low-temperature stress in both monocotyledonous and dicotyledonous plants, mainly by maintaining cell membrane stability and protecting photosynthetic pigments [32,33]. Recent studies have shown that the application of SiNPs can alleviate abiotic stress in plants [34,35]. In rice, available studies have mainly focused on the effects of SiNPs on heavy metal toxicity and other stress conditions, whereas their role in regulating low temperature tolerance remains poorly understood. Therefore, we hypothesized that the application of SiNPs could mitigate the negative effects of low-temperature stress on rice seedlings. To test this hypothesis, we conducted an experiment to investigate the effects of SiNPs and Ion-Si on the agronomic traits, osmolyte, antioxidant enzymes, chlorophyll content, and yield of rice seedlings under low-temperature stress. In this study, the regulation of low-temperature tolerance in rice seedlings by foliar application of ionic silicon (Na_2_SiO_3_: 100 mg/L) and SiNPs (SiO_2_: 100 mg/L) was investigated. Furthermore, the physiological characteristics, growth, and yield differences of rice under cold stress at the seedling stage were elaborated, providing a theoretical basis for enhancing the cold tolerance of rice through ionic silicon and silicon nanoparticles.

## 2. Results

### 2.1. Effects of Exogenous Silicon on Rice Growth Under Low Temperature Conditions

**Figure 1:**
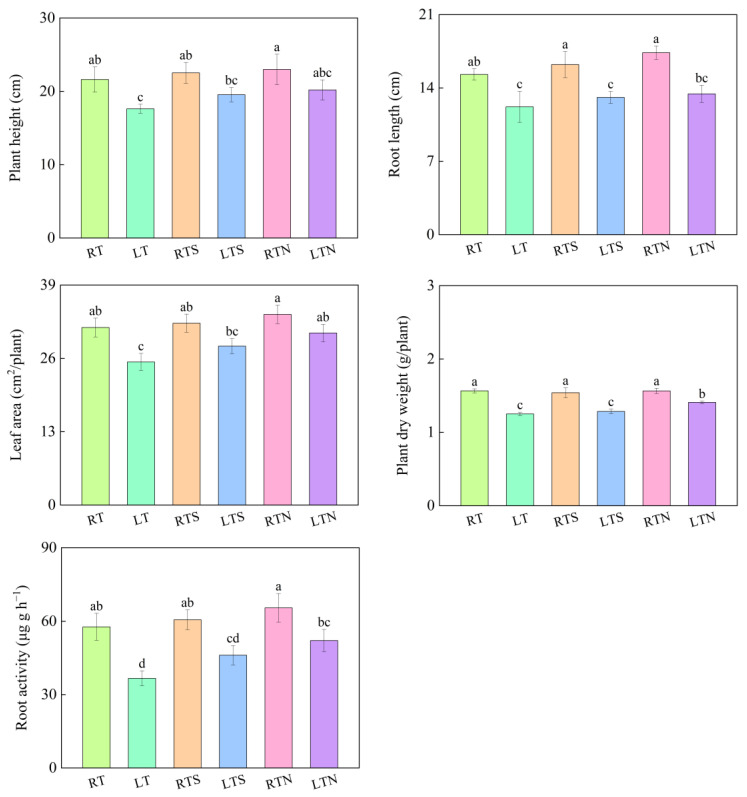
Effects of exogenous silicon on rice seedling growth under low temperature stress. Values were presented as means ± SD, *n* = 3. Different letters indicate statistically significant differences (*p* < 0.05).

Low temperature stress inhibited the growth of rice seedlings and reduced plant height, root length, leaf area, dry weight, and root activity (Figure 1). Under low temperature stress, exogenously applied Ion-Si and SiNPs improved rice growth. Nanoparticle treatment was more effective than ionic silicon formulation in promoting growth. Compared with RT treatment, LT treatment reduced plant height, root length, leaf area, dry weight, and root activity by 18.47%, 20.20%, 19.40%, 20.10%, and 36.50%, respectively. Compared with the RTS treatment, the LTS treatment reduced rice plant height, root length, leaf area, dry weight, and root vigor by 13.21%, 19.34%, 12.61%, 15.56%, and 23.89%, respectively. Compared with the RTN treatment, the LTN treatment reduced rice plant height, root length, leaf area, dry weight, and root vigor by 12.17%, 21.05%, 9.77%, 10.01%, and 20.47%, respectively. These results indicate that SiNPs maintained rice growth more effectively than Ion-Si under low-temperature stress.

### 2.2. Effects of Exogenous Silicon on Photosynthetic Parameters of Rice Under Low Temperature Conditions

**Figure 2:**
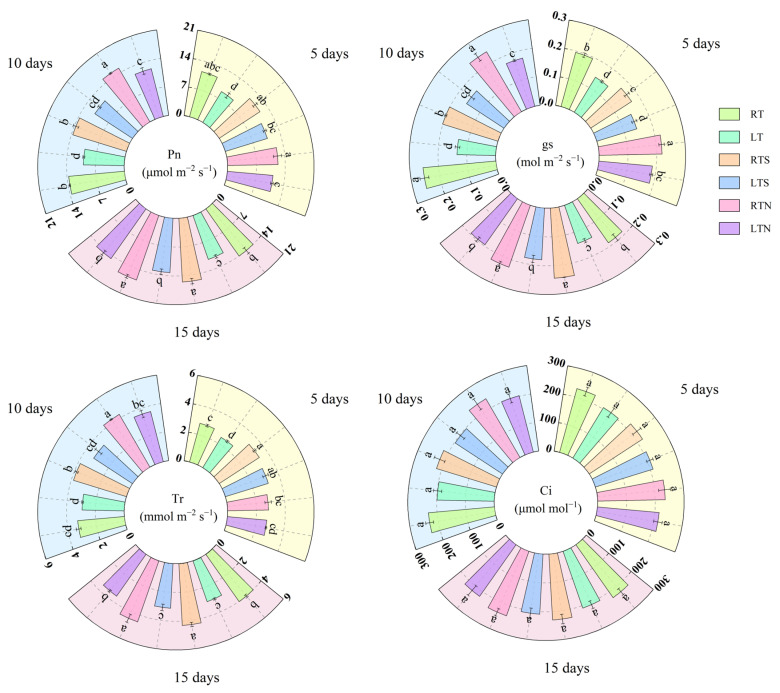
Effect of exogenous silicon on photosynthesis-related parameters of rice under low-temperature stress. Values were presented as means ± SD, *n* = 3. Different letters indicate statistically significant differences (*p* < 0.05).

Low-temperature stress affected the photosynthetic capacity of rice leaves (Figure 2). Compared with the corresponding normal-temperature treatments, low-temperature stress reduced Pn, Tr, Ci, and gs to varying degrees. After 5 days of treatment, compared with RT, Pn, Tr, Ci, and gs under LT decreased by 22.82%, 11.58%, 10.62%, and 27.00%, respectively. Compared with RTS, Pn, Tr, Ci, and gs under LTS decreased by 9.59%, 6.21%, 5.65%, and 14.33%, respectively. Compared with RTN, Pn, Tr, Ci, and gs under LTN decreased by 9.23%, 3.45%, 8.01%, and 14.85%, respectively. After 15 days of treatment, compared with RT, Pn, Tr, Ci, and gs under LT decreased by 19.48%, 25.37%, 7.41%, and 26.54%, respectively. Compared with RTS, Pn, Tr, Ci, and gs under LTS decreased by 14.87%, 27.95%, 8.42%, and 25.85%, respectively. Compared with RTN, Pn, Tr, Ci, and gs under LTN decreased by 11.01%, 20.71%, 7.21%, and 13.65%, respectively. Pn, Tr, and gs showed significant differences among treatments at the same sampling time, whereas Ci did not show significant differences between silicon-treated and untreated plants under either normal-temperature or low-temperature conditions. Under low-temperature conditions, silicon application increased Pn, Tr, and gs compared with LT, with SiNPs showing a stronger effect than Ion-Si. However, the changes in Ci were not statistically significant. Therefore, the alleviating effect of exogenous silicon on low-temperature-induced photosynthetic inhibition was mainly reflected in the improvement of Pn, Tr, and gs rather than Ci.

### 2.3. Effects of Exogenous Silicon on Chlorophyll Content in Rice Under Low Temperature Conditions

**Figure 3:**
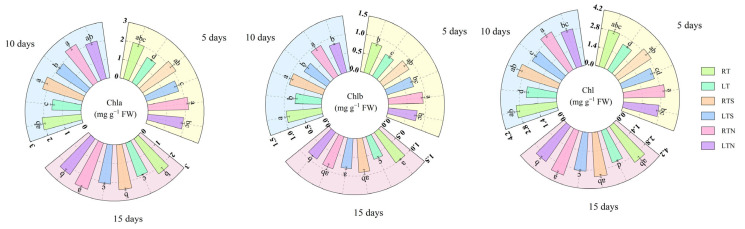
Effects of exogenous silicon on chlorophyll content in rice under low temperature stress. Values were presented as means ± SD, *n* = 3. Different letters indicate statistically significant differences (*p* < 0.05).

Low temperature stress reduced the chlorophyll content in rice leaves (Figure 3). After 5 days of treatment, compared with the RT treatment, the Chla, Chlb, and Chl in rice leaves under LT treatment decreased by 21.67%, 17.25%, and 20.38%, respectively; compared with the RTS treatment, the Chla, Chlb, and Chl in rice leaves under LTS treatment decreased by 15.64%, 10.79%, and 14.27%, respectively; compared with the RTN treatment, the Chla, Chlb, and Chl in rice leaves under LTN treatment decreased by 11.89%, 16.81%, and 13.37%, respectively. After 15 days of treatment, compared with the RT treatment, the Chla, Chlb, and Chl contents in rice leaves decreased by 17.43%, 35.58%, and 22.92%, respectively, under the LT treatment. Compared with the RTS treatment, the Chla, Chlb, and Chl contents decreased by 12.81%, 10.81%, and 12.26%, respectively, under the LTS treatment. Compared with the RTN treatment, the Chla, Chlb, and Chl contents decreased by 10.26%, 3.72%, and 8.57%, respectively, under the LTN treatment. These results indicate that the application of exogenous silicon alleviated the low-temperature-induced inhibition of photosynthetic pigment synthesis in rice leaves, with SiNPs outperforming Ion-Si in the regulation of photosynthetic pigments.

### 2.4. Effects of Exogenous Silicon on Enzyme Activities Related to Glucose Metabolism in Rice Under Low Temperature Conditions

**Figure 4:**
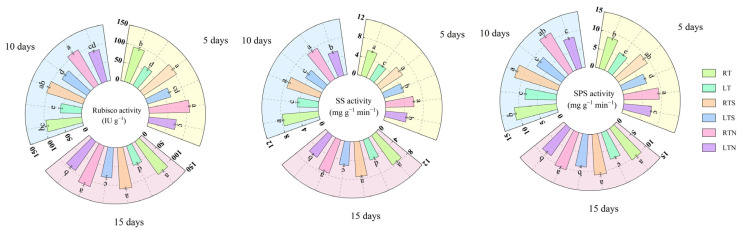
Effects of exogenous silicon on activities of enzymes related to sugar metabolism in rice under low temperature stress. Values were presented as means ± SD, *n* = 3. Different letters indicate statistically significant differences (*p* < 0.05).

Low temperature stress reduced the activities of enzymes related to sugar metabolism in rice (Figure 4). Within 5–15 days, compared with RT treatment, after LT treatment, the Rubisco activity of rice leaves decreased by 37.67–48.46%, SS activity decreased by 31.69–41.78%, and SPS activity decreased by 25.63–30.07%. Compared with RTS treatment, after LTS treatment, the Rubisco activity of rice leaves decreased by 21.28–38.14%, SS activity decreased by 24.24–36.76%, and SPS activity decreased by 19.33–33.61%. Compared with RTN treatment, after LTN treatment, the Rubisco activity of rice leaves decreased by 17.85–32.62%, SS activity decreased by 22.47–29.19%, and SPS activity decreased by 14.57–28.01%. These results indicate that the application of exogenous silicon alleviated the low-temperature-induced activities of enzymes related to sugar metabolism in rice, among which SiNPs outperformed Ion-Si in regulating the activities of enzymes related to sugar metabolism.

### 2.5. Effects of Exogenous Silicon on Antioxidant Enzyme Activities in Rice Under Low Temperature Conditions

**Figure 5:**
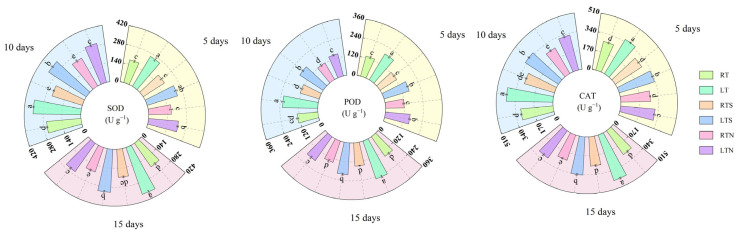
Effect of exogenous silicon on antioxidant enzyme activities in rice under low temperature stress. Values were presented as means ± SD, *n* = 3. Different letters indicate statistically significant differences (*p* < 0.05).

Low-temperature stress increased antioxidant enzyme activities in rice leaves (Figure 5). After 5 days of treatment, compared with RT, the activities of SOD, POD, and CAT under LT increased by 48.15%, 45.69%, and 35.47%, respectively. Compared with RTS, the activities of SOD, POD, and CAT under LTS increased by 25.92%, 33.41%, and 16.95%, respectively. Compared with RTN, the activities of SOD, POD, and CAT under LTN increased by 30.41%, 33.14%, and 15.47%, respectively. After 15 days of treatment, compared with RT, the activities of SOD, POD, and CAT under LT increased by 71.54%, 67.23%, and 64.12%, respectively. Compared with RTS, the activities of SOD, POD, and CAT under LTS increased by 53.77%, 36.42%, and 31.24%, respectively. Compared with RTN, the activities of SOD, POD, and CAT under LTN increased by 34.82%, 29.45%, and 25.74%, respectively. These results indicate that low-temperature stress induced antioxidant enzyme activities in rice leaves. However, under low-temperature conditions, the activities of SOD, POD, and CAT in silicon-treated plants were generally lower than those in LT plants, suggesting that silicon application may have reduced the oxidative pressure caused by low-temperature stress. Therefore, exogenous silicon alleviated low-temperature-induced oxidative stress rather than simply further increasing antioxidant enzyme activities, with SiNPs showing a stronger alleviating effect than Ion-Si.

### 2.6. Effect of Exogenous Silicon on Reactive Oxygen Species (ROS) in Rice Under Low Temperature Conditions

**Figure 6:**
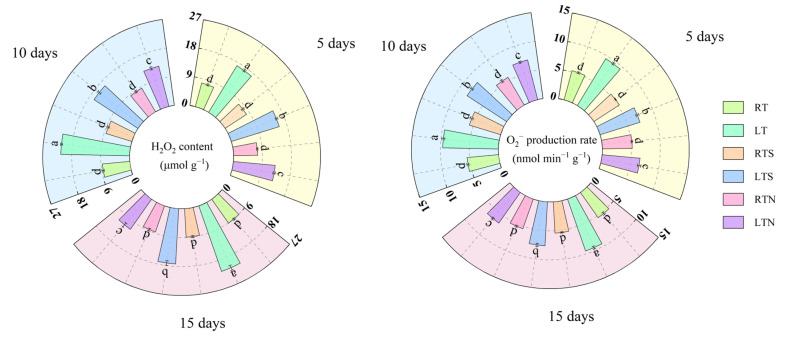
Effect of exogenous silicon on ROS in rice under low temperature stress. Values were presented as means ± SD, *n* = 3. Different letters indicate statistically significant differences (*p* < 0.05).

Low temperature stress increased the accumulation of ROS in rice (Figure 6). During 5–15 days, compared with the RT treatment, after the LT treatment, the H_2_O_2_ content increased by 75.82–81.46%, and the O_2_^−^ production rate increased by 122.45–148.79%; compared with the RTS treatment, after the LTS treatment, the H_2_O_2_ content increased by 37.56–44.93%, and the O_2_^−^ production rate increased by 93.46–97.41%; compared with the RTN treatment, after the LTN treatment, the H_2_O_2_ content increased by 19.45–27.12%, and the O_2_^−^ production rate increased by 33.49–74.95%. These results demonstrate that exogenous silicon application alleviated low-temperature-induced ROS accumulation in rice leaves, and SiNPs were more effective than Ion-Si. The lower ROS accumulation in silicon-treated plants may partly explain why their antioxidant enzyme activities were not as strongly induced as those in LT plants.

### 2.7. Effect of Exogenous Silicon on Osmotic Regulation Substances in Rice Under Low Temperature Conditions

**Figure 7:**
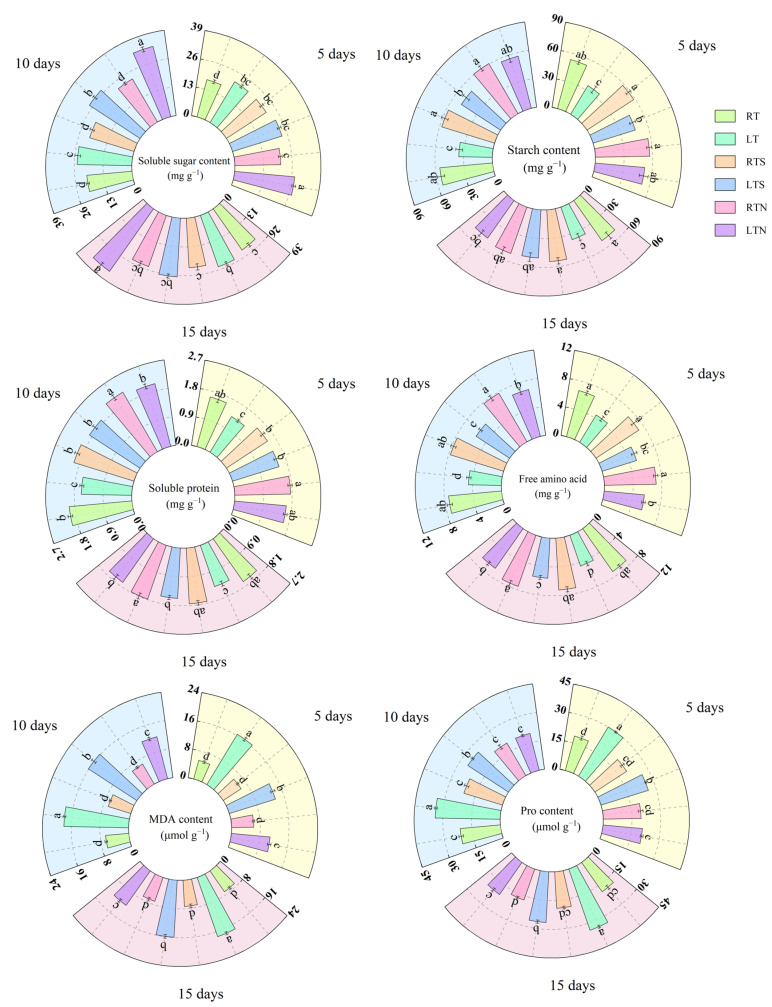
Effects of exogenous silicon on osmotic adjustment substances in rice under low temperature stress. Values were presented as means ± SD, *n* = 3. Different letters indicate statistically significant differences (*p* < 0.05).

Low temperature stress affected the changes in osmotic adjustment substances in rice (Figure 7). Compared with normal temperature, low temperature stress increased the contents of soluble sugar, MDA, and proline, while decreasing the contents of starch, soluble protein, and free amino acids. After 5 days of treatment, compared with RT treatment, the contents of soluble sugar, MDA, and proline under LT treatment increased by 23.54%, 205.86%, and 61.07%, respectively, while starch, soluble protein, and free amino acids decreased by 34.04%, 18.79%, and 32.89%, respectively. Compared with RTS treatment, the contents of soluble sugar, MDA, and proline under LTS treatment increased by 8.72%, 126.54%, and 30.48%, respectively, while starch, soluble protein, and free amino acids decreased by 20.84%, 1.41%, and 29.78%, respectively. Compared with RTN treatment, the contents of soluble sugar, MDA, and proline under LTN treatment increased by 31.84%, 75.25%, and 4.84%, respectively, while starch, soluble protein, and free amino acids decreased by 8.52%, 6.42%, and 22.05%, respectively.

After 15 days of treatment, compared with the RT treatment, the contents of soluble sugar, MDA, and proline increased by 7.92%, 145.51%, and 80.45%, respectively, following the LT treatment, while starch, soluble protein, and free amino acids decreased by 29.52%, 16.74%, and 37.89%, respectively. Compared with the RTS treatment, the contents of soluble sugar, MDA, and proline increased by 18.45%, 117.54%, and 39.10%, respectively, following the LTS treatment, while starch, soluble protein, and free amino acids decreased by 7.12%, 9.79%, and 22.90%, respectively. Compared with the RTN treatment, the contents of soluble sugar, MDA, and proline increased by 41.17%, 66.45%, and 19.46%, respectively, following the LTN treatment, while starch, soluble protein, and free amino acids decreased by 6.31%, 6.33%, and 10.52%, respectively. These results further indicated that exogenous silicon enhanced the low-temperature-induced osmotic adjustment capacity, with SiNPs outperforming Ion-Si in osmotic adjustment.

### 2.8. Effect of Exogenous Silicon on Rice Yield Under Low Temperature Conditions

As shown in Table 1, low-temperature stress inhibited rice yield, whereas exogenous silicon application alleviated the yield reduction. Compared with RT, LT reduced the effective panicle number, spikelets per panicle, seed setting rate, and yield by 13.22%, 9.46%, 6.47%, and 20.88%, respectively, while the 1000-grain weight increased by 8.22%. Compared with RTS, LTS reduced the effective panicle number, spikelets per panicle, seed setting rate, and yield by 9.87%, 6.28%, 3.15%, and 9.95%, respectively, while the 1000-grain weight increased by 5.86%. Compared with RTN, LTN reduced the effective panicle number, spikelets per panicle, seed setting rate, and yield by 6.88%, 2.69%, 2.09%, and 7.69%, respectively, while the 1000-grain weight increased by 3.44%. These results indicate that exogenous silicon alleviated low-temperature-induced yield loss in rice, with SiNPs showing a stronger protective effect than Ion-Si.

### 2.9. Correlation Analysis

**Figure 8:**
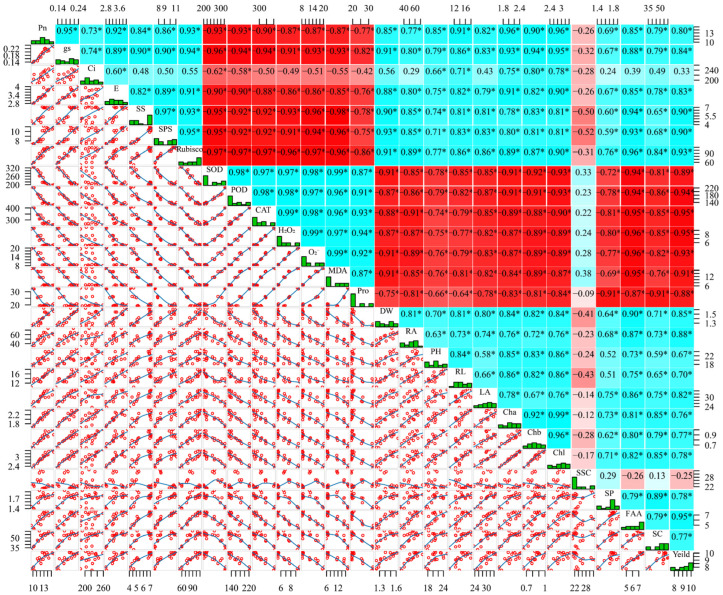
Correlation analysis of various indicators. * indicates *p* < 0.05. DW: dry weight; RA: root activity; PH: plant height; RL: root length; LA: leaf area; SSC: soluble sugar; SP: soluble protein; FAA: free amino acid; SC: starch.

The results of the correlation analysis indicate that photosynthesis-related parameters are positively correlated with sugar-metabolism-related enzymes, rice growth, photosynthetic pigments, and yield, and negatively correlated with antioxidant-related enzymes and ROS accumulation (Figure 8). Among these, the correlation results for yield are consistent with the changes in photosynthetic parameters.

### 2.10. Principal Component Analysis

**Figure 9:**
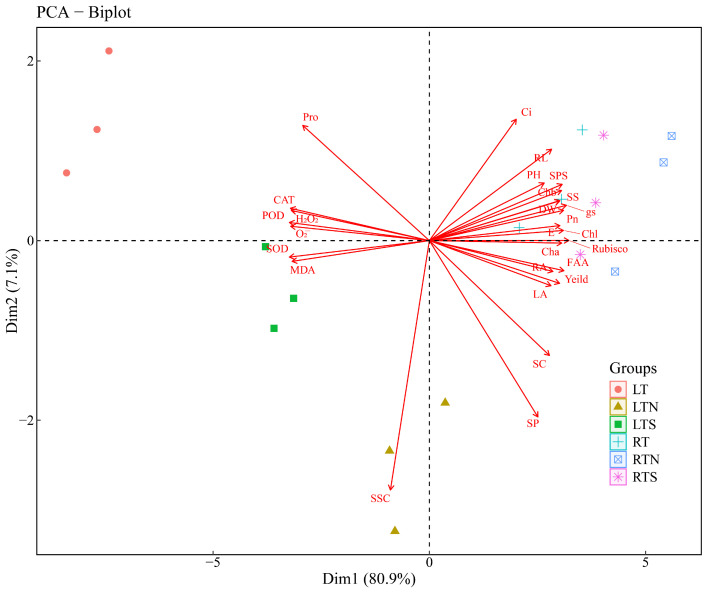
Principal component analysis of various indicators.

The results of principal component analysis (PCA) showed that the first two axes explained 88.0% of the total observed variation (Figure 9). Principal component 1 (80.9%) was related to Rubisco, Chl, Pn, FAA, and Yield, while principal component 2 (10.0%) was related to Pro, Ci, and RL.

## 3. Discussion

Plant phenotype is one of the most direct indicators of stress responses. Previous studies have shown that crops exposed to low temperatures for a long time are close to death [36]. The study by He et al. showed that low temperature stress significantly inhibited the growth of tomato seedlings, manifested as reduced leaf area and inhibited root development [37]. Previous studies have reported that K_2_SiO_3_ application can improve plant morphological traits, promote growth and development, and increase leaf area [38]. Both ionic silicon and SiNPs effectively mitigate growth inhibition in tomato seedlings caused by low temperature stress. Compared with seedlings exposed solely to LT stress, silicon-treated plants maintained dark green leaves, showed better development, and exhibited more vigorous root growth [37]. Previous studies have shown that rice seedling growth is markedly inhibited when the average temperature drops below 12 °C [39]. However, available literature on the effects of ionic silicon and nano-silicon on rice seedlings exposed to low-temperature stress is limited. The root system of a plant is the organ that absorbs the largest proportion of water and mineral nutrients, and its growth status and activity directly affect the development of the aboveground parts. The results of this study indicate that the application of Ion-Si and SiNPs treatments effectively alleviated the inhibitory effects of low-temperature stress on the growth of rice seedlings. These treatments improved plant height, root length, leaf area, dry weight, and root activity in rice, with SiNPs being more effective than Ion-Si in maintaining rice growth under low-temperature stress. Silicon can improve plant phenotypes by protecting cell ultrastructure (including chloroplasts, vacuoles, and nuclei) and maintaining the stability and integrity of cell walls [40]. Silicon application also increased plant height and biomass of cilantro while reducing cell death [23]. The results of this study are consistent with previous findings that low temperature stress significantly inhibited crop growth and impaired normal plant development. However, the application of ionic silicon and SiNPs promoted the recovery of rice.

Photosynthesis is the fundamental biosynthetic process by which plants obtain the energy and organic matter necessary for growth and development. Photosynthesis requires light; however, excessive light energy absorbed by pigment molecules can disrupt the oxidative balance within the plant, thereby inhibiting photosynthesis, particularly in C3 plants [41]. Nitrogen contributes to the composition of photosynthetic pigments, photosynthetic enzymes, and chloroplasts [42]. Chlorophyll is the primary photosynthetic pigment in plants, involved in the absorption, transfer, distribution, and conversion of light energy, directly affecting the plant’s efficiency in utilizing light energy and its accumulation of dry matter [43]. In this study, changes in chlorophyll content were consistent with the trend of rice growth. This indicates that low temperature weakens the leaf’s anti-senescence ability and reduces chlorophyll content. In this study, with the increase in stress days, chlorophyll content decreased under low-temperature stress, whereas the application of exogenous Si could alleviate this phenomenon. This is because Ion-Si and SiNPs enhanced the cellular structure of mesophyll, spongy tissue, and palisade tissue, maintaining chloroplast integrity under low-temperature stress. They increased chlorophyll content by alleviating oxidative damage [44]. Both Ion-Si and SiNPs improved chlorophyll content in rice under low temperature stress, with SiNPs showing a greater effect than Ion-Si. The stronger effect of SiNPs may be related to their specific physicochemical properties, such as high surface area, better adhesion and retention on leaf surfaces, and potential gradual release of plant-available silicon. These properties may enhance the protective effect of silicon on chlorophyll stability and photosynthetic performance under low temperature stress. Previous studies have also reported that SiNPs can improve photosynthetic pigment content in crops under stress conditions [45]. Future studies should include carotenoids and related photoprotective pigments to further clarify the mechanism.

Photosynthesis is the basis for crop biomass accumulation and yield formation. In this study, the changes in photosynthesis-related parameters were consistent with the trend of dry matter accumulation. Compared with normal temperature treatment, low temperature stress reduced the Pn, gs, E, and Ci of rice, while exogenous silicon improved rice photosynthesis. Photosynthesis is an important process for material accumulation, with over 95% of dry matter produced through photosynthesis. This study also confirmed this view, showing a significant positive correlation between photosynthetic parameters and dry matter. Under low temperature stress, SiNPs outperformed Ion-Si in improving photosynthetic capacity, with higher Pn, gs, E, and Ci. Haghighi and Pessarakli also reported similar findings, observing that SiNPs treatment enhanced chlorophyll synthesis and photosynthetic capacity in tomato seedlings [46]. Furthermore, a possible mechanism by which silicon protects photosynthetic pigments under stress is related to its ability to form a bilayer membrane on the cell wall, thereby helping to maintain cell structural stability [47].

Rubisco is a key enzyme affecting plant photosynthesis. Rubisco catalyzes the carboxylation and oxygenation of ribulose-1,5-bisphosphate (RuBP) and controls photosynthetic carbon metabolism and photorespiration in plants [48]. Relevant studies have shown that Rubisco activity is significantly reduced under low-temperature stress, which may be due to an increase in chloroplast protrusions and the release of Rubisco-containing vesicles (Rubisco-containing bodies), representing one of the pathways for Rubisco export from chloroplasts [49]. In this study, low-temperature stress decreased Rubisco activity, while exogenous silicon treatment increased Rubisco activity, with SiNPs showing a better improvement effect than Ion-Si. This is consistent with the findings of Pereira et al., who found that silicon application increased Rubisco activity related to silicon-induced regulation of photosynthesis [50].

Carbohydrates are vital to living organisms, directly participating in energy metabolism, and playing a key role in plant stress responses [51]. The cold tolerance of plants is positively correlated with soluble sugar content. Soluble sugars, as respiratory substrates, generate redox potential for proline synthesis through oxidative phosphorylation. They also act as osmotic regulators, increasing cytosolic concentration and protecting cytoplasmic colloids [52]. In this study, low temperature stress significantly increased soluble sugar content. The application of exogenous silicon further elevated its content, promoting translocation from roots to shoots to provide energy and osmotic regulation, thereby improving plant water relations under stress [53]. Under low temperature stress, exogenous silicon increased the activities of SPS and SS. This indicates that sucrose biosynthesis was induced via the SS/SPS pathway to protect cells under low temperature stress, while simultaneously reducing the accumulation and transport of photosynthates. The application of Ion-Si and SiNPs enhanced the activities of key sucrose-metabolizing enzymes, involved in carbon flux partitioning and photosynthate transport, while stabilizing cellular metabolism [54]. This effect may be attributed to the stress-protective role of silicon rather than a direct stimulation of enzyme activity, because silicon can reduce ROS-induced damage, maintain membrane and chloroplast integrity, and provide a more favorable metabolic environment for Rubisco, SS, and SPS under low-temperature stress. Under room-temperature conditions, these enzymes may already be maintained at normal physiological levels, which could explain the absence of significant differences among RT, RTS, and RTN after 15 days of treatment. Compared with Ion-Si, SiNPs further promoted the transport of photosynthetic products, alleviated the negative feedback mechanism effects, and enhanced efficiency under low-temperature stress [55]. The stronger effect of SiNPs may be related to their physicochemical properties and interaction with plant tissues, although this mechanism requires further verification.

ROS can integrate environmental stress signals to activate gene expression in response to stress, thus achieving a balance between plant defense and growth [9]. Stress induces the overproduction of ROS (including O_2_^−^ and H_2_O_2_) in plants, leading to increased oxidation and soluble protein concentration, which is conducive to enhancing plant stress tolerance but also affects normal plant physiological processes [56]. Proline is an amino acid that plays a very beneficial role in plants, helping to stabilize subcellular structures, scavenge free radicals, and buffer cellular antioxidant potential under various stress conditions. Free amino acids in plants are the basic units of proteins and precursors of functional compounds, and they are transported within the plant in the form of nitrogen assimilates. Low temperature stress disrupts cellular ROS homeostasis, triggering ROS activation of antioxidant enzymes and regulating ROS-responsive gene expression [9]. Antioxidant enzymes (SOD, POD, CAT) can scavenge excess ROS. The higher SOD, POD, and CAT activities under LT than under RT may represent an adaptive defense response to excessive ROS accumulation induced by low-temperature stress. This study showed that low temperature stress increased proline and MDA content. Exogenous silicon reduced H_2_O_2_ and MDA content and increased soluble protein and free amino acid content in rice seedlings, while moderating the low temperature-induced increase in antioxidant enzyme activities, thereby alleviating stress damage. This indicates that both Ion-Si and SiNPs enhance plant stress tolerance by strengthening antioxidant capacity and inhibiting lipid peroxidation. Under low temperature conditions, the relatively lower antioxidant enzyme activities in silicon-treated plants compared with LT may reflect reduced oxidative pressure rather than weakened antioxidant defense. Compared with Ion-Si, SiNPs more effectively reduced ROS accumulation and lipid peroxidation, which may explain their stronger ability to mitigate oxidative damage under low temperature stress. SiNPs enhanced the enzymatic ROS scavenging system and potential non-enzymatic antioxidant pathways under low-temperature stress [57].

As a thermophilic crop, the growth and development of rice can be adversely affected by low-temperature stress [2]. The results of this study indicate that low-temperature stress affects rice yield and its components, such as the number of grains per panicle, thousand-grain weight, and seed setting rate. Relevant studies have shown that the earlier the stress occurs after germination, the greater its impact on the seed setting rate. The effect of stress on the number of solid particles attached to the primary and secondary branches is roughly the same as its effect on the seed setting rate, which further clarifies that the reduction in seed setting rate caused by low temperature stress during early irrigation is the main reason for yield reduction. The results of this study indicate that exogenous silicon applied after low temperature stress can, to some extent, mitigate the adverse effects of low temperature on rice yield. Application of Ion-Si and SiNPs can withstand low temperature stress, thereby increasing rice yield. SiNPs were more effective than Ion-Si in maintaining rice yield, mainly by increasing the effective panicle number, grains per panicle, and seed setting rate under low-temperature stress. The increase in 1000-grain weight under low-temperature stress may be related to source–sink balance and a compensatory effect during grain filling. Low temperature stress reduced the effective panicle number, spikelets per panicle, and seed setting rate, which may have decreased the overall sink size. As a result, the remaining grains may have received relatively more assimilates, leading to a higher individual grain weight. However, this increase in 1000-grain weight did not compensate for the reductions in other yield components, and the final yield was still decreased under low temperature stress. Therefore, the higher 1000-grain weight under low temperature stress does not indicate that low temperature promoted yield formation.

It should be noted that this study focused mainly on the physiological and yield responses of rice to Ion-Si and SiNPs under low-temperature stress. Plant silicon content was not directly measured, and the internalization, transport, and distribution of SiNPs in rice tissues were not observed. Therefore, the present results cannot determine whether the effects of SiNPs were caused by direct nanoparticle uptake, dissolution into plant-available silicon species, retention on leaf surfaces, or their combined effects. Future studies should quantify silicon accumulation in different rice tissues and use techniques such as ICP-OES/ICP-MS, TEM or SEM-EDS, and labeled SiNPs to clarify the uptake and transport mechanisms of SiNPs in rice.

## 4. Materials and Methods

### 4.1. Test Materials

The experimental rice was the commonly promoted variety Jikedao 654 in Jilin City, China. This variety is a rice variety with excellent traits and strong disease resistance, classified as a mid-late maturing rice variety (maturing within about 150 days).

### 4.2. Experimental Design

The experiment was conducted from May to September 2025 in the experimental field of Jilin Agricultural Science and Technology College. Pre-germination treatment was carried out on 8 April. Rice was sown on 11 April and cultivated in a greenhouse until 12 May. On 12 May, the rice seedlings were treated and transplanted into growth chambers with a light intensity of 10,000 lx, a photoperiod of 12/12 h, and a relative humidity of 50%. The temperature was set at room temperature (RT) and low temperature (LT), with six treatments in total, as shown in Table 2. Ionic silicon was supplied as sodium silicate (Ion-Si, analytical grade), which was purchased from Tianjin Baishi Chemical Reagent Co., Ltd. (Tianjin, China). Silicon nanoparticles (SiNPs, 99%) were obtained from Beijing Solarbio Science & Technology Co., Ltd. (Beijing, China). The SiNP stock suspension had an original concentration of 2.00 mol·L^−1^. The SiNPs had an average particle size of approximately 20 nm and a zeta potential of −31.329 ± 0.23 mV. The silicon treatment solutions were prepared using deionized water as the solvent, and no buffer solution was used. Sodium silicate was dissolved directly in deionized water to obtain the required working concentrations. The SiNPs stock suspension was fully mixed by vortexing before dilution and application. All solutions were freshly prepared before use. The control plants were sprayed with the same volume of deionized water. Because the original experiment was conducted as a pot experiment in growth chambers, the silicon application rate was controlled by working concentration rather than by field application rate. Foliar application was performed using a hand sprayer until the leaf surfaces were uniformly wetted without visible runoff. To estimate the actual spray volume, a post-experiment calibration was conducted using the same sprayer and the same spraying endpoint. Because Ion-Si and SiNPs differ in chemical composition and molecular weight, the actual amount of elemental silicon supplied by the two sources may differ. This difference was considered when interpreting the physiological effects of the two silicon sources.

After 15 days of treatment, on 27 May, seedlings from the six treatments were transplanted into the experimental field. The transplanting spacing for rice was 30 cm between rows and 20 cm between hills, with 3 seedlings per hill. Water management for each treatment adopted single-row irrigation and drainage. Fertilizer application rates followed the traditional fertilization practices of local farmers. The application rate of phosphate fertilizer (P_2_O_5_) was 75 kg ha^−1^, potassium fertilizer (K_2_O) was 150 kg ha^−1^, and nitrogen fertilizer (46% urea) was applied at 150 kg ha^−1^ of pure N. On May 23 (before harrowing), 40% of N, 100% of P_2_O_5_, and 70% of K_2_O were applied as basal fertilizer. On 5 June, 30% of N was applied as tillering fertilizer, and the remaining 30% of N and 30% of K_2_O were applied as panicle fertilizer on 1 July. For other field management methods, refer to technology model for lodging resistance, senescence prevention and high yield of japonica rice in central Songnen Plain.

### 4.3. Measurement Indicators and Methods

#### 4.3.1. Determination of Rice Growth Indicators

Fifteen days after treatment, rice seedlings were sampled. Three rice seedlings with similar growth status were selected from each treatment to measure plant height, root length, and leaf area. Plant samples were dried at 105 °C for 30 min, then dried at 80 °C for 3 days to measure total plant dry weight.

Root vitality was determined using a Root Vitality Assay Kit (Shanghai Yuanye Biological Technology Co., Ltd., Shanghai, China). Briefly, 0.5 g of fresh clean root samples were incubated in 2,3,5-triphenyltetrazolium chloride (TTC) solution at 37 °C for 1 h. After grinding and extraction with ethyl acetate, the supernatant was collected and then measured according to the manufacturer’s instructions.

#### 4.3.2. Measurement of Photosynthetic Characteristics and Chlorophyll Content

At 5, 10, and 15 days after treatment, three rice plants with similar growth status were selected from each treatment. The uppermost fully expanded leaf of each selected plant was used for the measurement of photosynthetic parameters. From 8:00 to 11:00 a.m., net photosynthetic rate (Pn), stomatal conductance (gs), transpiration rate (Tr), and intercellular CO_2_ concentration (Ci) were measured using a portable photosynthesis system (Li-6800, Li-COR, Lincoln, NE, USA). The light intensity in the leaf chamber was set to 1200 µmol·m^−2^·s^−1^. Rice leaves for all treatments were taken from the uppermost fully expanded leaves. Three repeated measurements were performed for each treatment.

Chlorophyll content in rice leaves was calculated using the 90% ethanol extraction method based on the absorbance at 649 nm and 665 nm [58]. The concentrations of chlorophyll a (Chla), chlorophyll b (Chlb), and total chlorophyll (Chl) are calculated using the following formulas: Chla = 13.95 ∗ A665 − 6.8 ∗ A649; Chlb = 24.96 ∗ A649 − 7.32 ∗ A665; Chl = Chla + Chlb.

The chlorophyll content was then expressed on a fresh-weight basis using the following equation:

Chlorophyll content (mg·g^−1^ FW) = C × V × D/(1000 × W)

where C is the chlorophyll concentration in the extract, V is the final extract volume in mL, D is the dilution factor, and W is the fresh weight of the leaf sample in g.

#### 4.3.3. Determination of Glucose Metabolism Enzyme Activity

At 5, 10, and 15 days after treatment, 30 representative rice plants with similar growth status were selected from each treatment. The samples from these plants were randomly divided into three groups and pooled within each group to form three biological replicates. These pooled samples were used for subsequent physiological and biochemical analyses. A fresh leaf sample of 0.2 g (with midrib removed) was taken, and 2 mL of extraction buffer (50 mmol·L^−1^ Hepes-NaOH, pH 7.5; 50 mmol·L^−1^ MgCl_2_; 2 mmol·L^−1^ EDTA; 0.2% BSA; 2% PVP) was added. The mixture was thoroughly ground into a homogenate on ice, then centrifuged at 4 °C and 12,000 rpm for 10 min in a high-speed refrigerated centrifuge. The supernatant was collected as the enzyme extract. A 50 µL aliquot of enzyme extract was mixed with the reaction solution containing 50 µL of 50 mmol·L^−1^ Hepes-NaOH buffer (pH 7.5), 20 µL of 50 mmol·L^−1^ MgCl_2_, 20 µL of 100 mmol·L^−1^ UDPG, and 20 µL of 100 mmol·L^−1^ fructose or fructose-6-phosphate. The mixture was incubated in a water bath at 30 °C for 30 min. The reaction was terminated by adding 200 µL of 2 mol·L^−1^ NaOH solution, and the mixture was then placed in a boiling water bath for 10 min. After cooling, 1.5 mL of 30% hydrochloric acid solution and 0.5 mL of 0.1% resorcinol solution were added. Color development was performed in an 80 °C water bath for 10 min. After cooling, the absorbance was measured at 480 nm. The activities of sucrose synthase (SS) and sucrose phosphate synthase (SPS) were expressed as the amount of sucrose or sucrose phosphate produced per unit time per unit mass of leaves and grains (mg·g^−1^·min^−1^) [59].

The Rubisco activity was determined using enzyme-linked immunosorbent assay (ELISA) with a kit produced by Jiangsu Edison Biotechnology Co., Ltd., (Yancheng, China) and each treatment was measured in triplicate.

#### 4.3.4. Determination of Antioxidant Enzyme Activity and Substance Content

SOD, POD, and CAT activities were determined according to Li et al. [60]. MDA content was measured using the thiobarbituric acid method [60]. The contents of H_2_O_2_ and O_2_^−^ were determined according to Zhang [61]. Proline content was determined according to Li [62].

Extraction was performed according to the method of Yoshida et al. [63]. After drying to constant weight, the samples were ground into a fine powder, then mixed with 80% ethanol, incubated in a water bath at 80 °C, cooled, and centrifuged. This extraction was repeated three times. The supernatants were combined and made up to 50 mL with distilled water for the determination of total soluble sugars [61]. Each treatment was measured in triplicate.

The residue remaining after extracting soluble total sugars was dried in an oven at 80 °C. Then, HClO_4_ was added to the dried residue for extraction. After centrifugation, the supernatant was decanted, and the extraction was repeated three times. The supernatants were combined and made up to 50 mL with distilled water for starch determination [64]. Each treatment was measured in triplicate.

A 0.2 g fresh sample was weighed, ground with 5 mL of 10% acetic acid, diluted to 20 mL with ammonia-free distilled water, and then filtered. A 1 mL aliquot of the filtrate was transferred into a test tube, followed by the addition of 3 mL of ninhydrin reagent, 1 mL of ammonia-free distilled water, and 0.1 mL of ascorbic acid. The test tube was placed in boiling water for 15 min and then cooled in cold water. After the solution turned bluish-purple, 60% ethanol was added, and the volume was adjusted to 20 mL. The absorbance was measured at 570 nm, and the free amino acid content was calculated accordingly [61]. Three replicate measurements were performed for each treatment.

A 0.2 g fresh sample was weighed, mixed with 5 mL of distilled water, and ground into a homogenate. The homogenate was centrifuged at 3000 rpm for 10 min, and the supernatant was collected for further analysis. A 1 mL aliquot of the supernatant was transferred into a test tube, followed by the addition of 5 mL of Coomassie Brilliant Blue reagent. The mixture was thoroughly mixed and allowed to stand for 2 min. The absorbance was then measured at 595 nm, and the soluble protein content was calculated [61]. Each treatment was measured in triplicate.

#### 4.3.5. Yield and Yield Components

During the mature stage, after removing border rows and off-type plants from each plot, an area of 5 m^2^ was harvested to calculate rice yield, with three replicates per treatment. The yield was then converted to rice grain yield at 14% moisture content. Nine representative rice plants were selected from each plot for plant trait analysis, measuring effective panicles per unit area, total grains per panicle, thousand-grain weight, and seed setting rate.

### 4.4. Statistical Analysis

Analysis of variance was performed using SPSS 24.0 software (SPSS Inc., Chicago, IL, USA), and multiple comparisons were conducted using Duncan’s multiple range test at *p* < 0.05. Data were further analyzed and graphed using Microsoft Excel 2023. Origin 2020 software was used for plotting. The “gpairs” package (version 1.4.0) in R version 4.4.3 was used for correlation analysis and plotting, and the “ggplot2” package (version 4.0.1) was used for principal component analysis and plotting.

## 5. Conclusions

Under low-temperature stress, Ion-Si and SiNPs effectively alleviated the growth inhibition of rice by increasing plant height, root length, leaf area, dry matter accumulation, and root activity. Silicon application also enhanced photosynthetic performance by increasing chlorophyll content, photosynthetic parameters, and Rubisco activity. Furthermore, Ion-Si and SiNPs regulated carbohydrate metabolism by increasing soluble sugar content and promoting the activity of sucrose-metabolizing enzymes, thereby contributing to osmotic adjustment, energy supply, and photoassimilate transport under low-temperature stress. Both forms of silicon enhanced the antioxidant defense system by increasing the activities of SOD, POD, and CAT, reducing the accumulation of H_2_O_2_ and MDA, and improving osmotic and metabolic stability, thus alleviating oxidative damage induced by low-temperature stress. Notably, SiNPs exhibited a stronger effect than Ion-Si in increasing chlorophyll content, photosynthetic capacity, Rubisco activity, carbohydrate metabolism, and antioxidant defense. In summary, SiNPs were more effective in maintaining the growth and final yield of rice seedlings. Overall, exogenous silicon enhances rice tolerance to stress by coordinately regulating growth, photosynthesis, carbon metabolism, antioxidant defense, and yield formation, with SiNPs showing a clear advantage in improving low-temperature stress. The results of this study will provide a reference for the regulation of SiNPs on the growth recovery of rice seedlings after low-temperature stress.

## Figures and Tables

**Table 1:** Effect of exogenous silicon on rice yield under low temperature stress.

Treatment	Effective Panicle Number (×10^4^/ha)	Spikelets per Panicle	Seed Setting Rate (%)	1000-Grain Weight (g)	Yield (t/ha)
RT	370.41 ± 1.70 a	122.24 ± 2.01 a	94.36 ± 0.78 a	23.5 ± 0.07 d	9.84 ± 0.21 a
LT	321.45 ± 4.58 d	110.67 ± 1.71 c	88.25 ± 1.05 c	25.43 ± 0.12 a	7.79 ± 0.19 c
RTS	372.65 ± 7.89 a	123.70 ± 2.46 a	94.46 ± 1.40 a	23.54 ± 0.07 d	9.86 ± 0.41 a
LTS	335.87 ± 3.35 c	115.93 ± 2.05 b	91.49 ± 0.49 b	24.92 ± 0.10 b	8.88 ± 0.28 b
RTN	371.86 ± 3.31 a	123.97 ± 2.18 a	94.8 ± 0.53 a	23.56 ± 0.04 d	9.92 ± 0.08 a
LTN	346.29 ± 2.87 b	120.63 ± 2.00 a	92.82 ± 0.41 ab	24.37 ± 0.04 c	9.15 ± 0.13 b

Note: Values were presented as means ± SD, *n* = 3. Different letters indicate statistically significant differences (*p* < 0.05).

**Table 2:** Definitions of different treatments.

Temperature Group	Treatment
RT	Spray distilled water + 26 °C/20 °C day/night temperature
LT	Spray distilled water + 14 °C/20 °C day/night temperature
RTS	Spray 100 mg/L Na_2_SiO_3_ + 26 °C/20 °C day/night temperature
LTS	Spray 100 mg/L Na_2_SiO_3_ + 14 °C/10 °C day/night temperature
RTN	Spray 100 mg/L SiO_2_-NPs + 26 °C/20 °C day/night temperature
LTN	Spray 100 mg/L SiO_2_ NPs + 14 °C/10 °C day/night temperature

## Data Availability

The original contributions presented in the study are included in the article; further inquiries can be directed to the corresponding authors.
